# Subcutaneous Emphysema, Pneumomediastinum, and Pneumorrhachis after Cocaine Inhalation

**DOI:** 10.1155/2015/134816

**Published:** 2015-07-08

**Authors:** Tuğba Atmaca Temrel, Alp Şener, Ferhat İçme, Gül Pamukçu Günaydın, Şervan Gökhan, Yavuz Otal, Gülhan Kurtoğlu Çelik, Ayhan Özhasenekler

**Affiliations:** ^1^Department of Emergency Medicine, Ankara Atatürk Training and Research Hospital, Üniversiteler Mahallesi Bilkent Caddesi No. 1, Çankaya, 06800 Ankara, Turkey; ^2^Department of Emergency Medicine, Faculty of Medicine, Yildirim Beyazit University, Üniversiteler Mahallesi Bilkent Caddesi No. 1, Çankaya, 06800 Ankara, Turkey

## Abstract

*Introduction*. The most prominent complications of cocaine use are adverse effects in the cardiovascular and central nervous systems. Free air in the mediastinum and subcutaneous tissue may be observed less frequently, whereas free air in the spinal canal (pneumorrhachis) is a very rare complication of cocaine abuse. In this report we present a case of pneumorrhachis that developed after cocaine use. *Case*. A 28-year-old male patient was admitted to the emergency department with shortness of breath, chest pain, and swelling in the neck and face which started four hours after he had sniffed cocaine. On physical examination, subcutaneous crepitations were felt with palpation of the jaw, neck, and upper chest area. Diffuse subcutaneous emphysema, pneumomediastinum, and pneumorrhachis were detected in the computed tomography imaging. The patient was treated conservatively and discharged uneventfully. *Discussion*. Complications such as pneumothorax, pneumomediastinum, and pneumoperitoneum that are associated with cocaine use may be seen due to increased intrathoracic pressure. The air then may flow into the spinal canal resulting in pneumorrhachis. Emergency physicians should know the possible complications of cocaine use and be prepared for rare complications such as pneumorrhachis.

## 1. Introduction

Cocaine leads to the most emergency department visits in the USA due to illicit drug use and it is the most frequently abused substance in Europe after cannabis [1]. Cocaine can be consumed in several ways such as smoking (crack) or sniffing or in an intravenous way. The mortality and morbidity associated with cocaine abuse usually occur due to acute cardiovascular or neurological complications [1].

The complications associated with inhaled cocaine use such as pneumothorax, pneumomediastinum, pneumopericardium, pneumoperitoneum, and pneumorrhachis are thought to occur due to barotrauma caused by increased intrathoracic pressure. Increased intrathoracic pressure is a result of either deep inhalation followed by the prolonged valsalva maneuver that is done by the individuals in order to augment absorption and enhance the desired effect of the drug or cough caused by the sniffed substance [2, 3].

In this paper we presented a case of subcutaneous emphysema, pneumomediastinum, and pneumorrhachis that developed after cocaine use.

## 2. Case

A 28-year-old male patient was admitted to the emergency department with shortness of breath, chest pain, and swelling in the neck and face which developed four hours after he had sniffed cocaine. The patient had a history of cocaine addiction; there was no history of other diseases, drugs, trauma, recent surgery, or air travel. The vital signs were blood pressure of 150/80 mmHg, pulse of 150 beats/min, body temperature of 37.8°C, and SpO2 of 96%. On his physical examination, the patient was conscious, oriented, and cooperative but seemed agitated; subcutaneous crepitations were palpated in the jaw, neck, and upper chest area; his skin was sweaty. Breath sounds were natural and equal bilaterally on auscultation; on cardiac examination heart sounds were rhythmic and tachycardic; there was no additional heart sound or murmur; no significant lateralized motor findings were detected on neurological examination. Other systemic physical examinations were normal. The electrocardiogram revealed sinus tachycardia. The patient was given 5 mg diazepam intravenously for agitation. The patient's blood pressure and tachycardia returned to normal, after his agitation declined.

**Figure 1:**
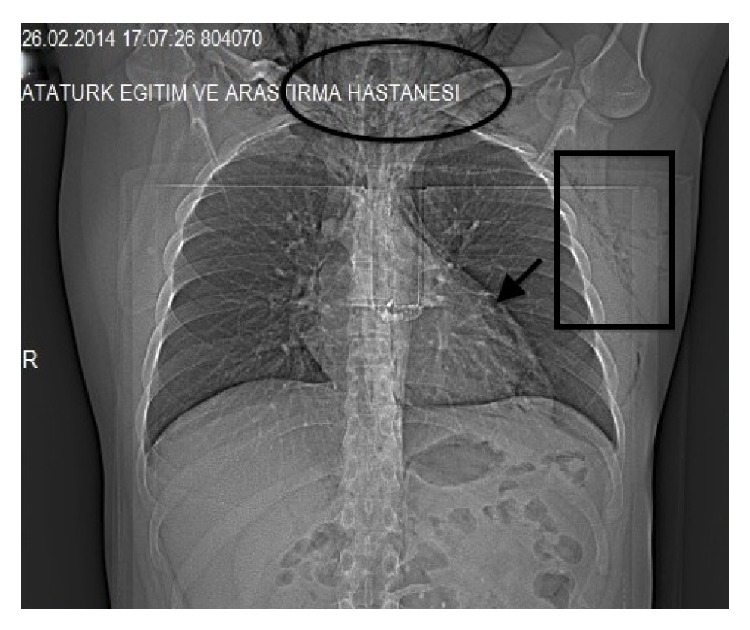
Subcutaneous air in the neck (oval) and in the axilla (rectangle) and air around the heart (arrow).

His chest X-ray revealed air in the subcutaneous tissue of the left axilla, the neck, and around the heart (Figure 1).

**Figure 2:**
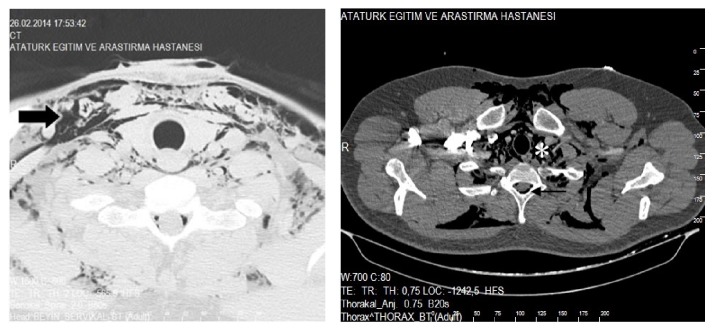
Diffuse subcutaneous emphysema (thick arrow), free air in the mediastinum (asterisk), and free air in the spinal canal (thin arrow).

When computed tomography (CT) imaging of the neck and chest was obtained, diffuse subcutaneous emphysema, pneumomediastinum, and pneumorrhachis were detected; no rib fractures were observed (Figure 2).

The patient was consulted with thoracic surgery and neurosurgery departments. During the follow-up in the emergency department, upon increase of dyspnea and swelling on the neck, the thoracic surgeon applied a skin incision over the suprasternal notch and provided free air drainage. Conservative follow-up by neurosurgery was recommended for pneumorrhachis. The patient was transferred to the intensive care unit of the thoracic surgery department. He was treated with oxygen and analgesics. The patient was discharged after 10 days of follow-up with no additional complications.

## 3. Discussion

The toxic effects of cocaine depend on the increased central and peripheral catecholamine activity. In our patient sinus tachycardia and agitation were observed and were treated with diazepam.

Cocaine inhalation has previously been linked to pneumomediastinum [3]. It occurs secondary to barotrauma, resulting in rupture of terminal alveoli into the lung interstitium and the dissection of air along the pulmonary vasculature toward the hilum and extravasation to mediastinum [3]. Over 90% of the cases present with chest pain. Neck pain, dyspnea, hoarseness, and dysphagia may also be seen. It may be associated with subcutaneous emphysema in 64% of the cases and pneumothorax in 19% of the cases [3]. Diagnosis is usually made with a posterior-anterior and lateral chest radiograph; computed tomography can help diagnosis [3]. Treatment is conservative (oxygen, analgesics) [3].

To our knowledge, this is the 3rd case in the medical literature reporting pneumorrhachis after cocaine use. The etiology of pneumorrhachis includes trauma (skull or spine injury), medical procedures (epidural anesthesia, lumbar puncture, and surgery), epidural abscess, and malignancies [4, 5]. It is found in combination with associated air distribution in other compartments and cavities of the body, particularly, in conjunction with pneumocephalus, pneumothorax, pneumomediastinum, pneumopericardium, or subcutaneous emphysema [6]. Diagnosis is usually made on chest computed tomography scan [4].

Pneumorrhachis is usually asymptomatic, does not tend to migrate, and is reabsorbed spontaneously and completely in several days without recurrence. Therefore, patients with pneumorrhachis are usually managed conservatively [6]. Same management is advised in spontaneous pneumomediastinum with and without pneumorrhachis [5].

The mechanism of cocaine-induced subcutaneous emphysema, pneumomediastinum, and pneumorrhachis is believed to be secondary to barotrauma caused by deep inhalation and valsalva maneuver done by abusers in order to increase uptake and the euphoriant effect of substance or cough triggered by the sniffed substance. Increased intra-alveolar pressure causes rupture of a distended alveolus into the lung interstitium, and air passes into the lung interstitium; air then migrates along the pulmonary vasculature toward the lung hilum and then to the posterior mediastinum and travels through the neural foramina into the epidural space [2]. Two additional mechanisms have been proposed: alveolar wall fragility caused by repeated cocaine sniffing and air leak from cocaine induced destructed nasopharyngeal structures [4].

In our case, we detected pneumorrhachis in addition to pneumomediastinum and subcutaneous emphysema. The patient was admitted to the intensive care unit and followed up conservatively, recovered, and was discharged uneventfully.

## 4. Conclusion

Since patients with complaints related to illegal substance use are often admitted to emergency departments, emergency physicians should know emergency management of their possible complications [7].
